# Recent Advances in the Devices for the Treatment of Chronic Obstructive Pulmonary Disease: A Review

**DOI:** 10.7759/cureus.49371

**Published:** 2023-11-24

**Authors:** Shivangi Jha, Dhurba Chandi

**Affiliations:** 1 Medicine, Jawaharlal Nehru Medical College, Datta Meghe Institute of Higher Education and Research, Wardha, IND; 2 Microbiology, Jawaharlal Nehru Medical College, Datta Meghe Institute of Higher Education and Research, Wardha, IND

**Keywords:** dpi, smi, mdi (metered-dose inhaler), asthma-copd overall syndrome, pulmonary disease

## Abstract

Chronic obstructive pulmonary disease or COPD has been known to adversely affect people’s quality of life. It influences a great number of individuals overall and is a main source of horribleness and mortality. It is associated with major healthcare and socioeconomic burdens. So, it is important to cure such types of diseases. This review article deals with the proper understanding of the newly developed devices and various advances taking place in the treatment of COPD. There are many new methods and procedures being developed recently for the cure or treatment of COPD, of which some are mentioned in the following review article. The articles also deal with the beneficial effects as well as the challenges faced during the use of those newly developed methods during the treatment of the disease. Various types of management of COPD are also mentioned in the article. This article also deals with the various new advances that are currently taking place in devices used in the therapy of COPD.

## Introduction and background

COPD, or chronic obstructive pulmonary disease, is currently the leading cause of death worldwide. The disease is marked by persistent respiratory symptoms and airflow restrictions. It is a treatable and preventable disease. It was ranked as the third most prevalent cause of death globally in or around 2016 and as the fourth most common cause of death in the United States in 2017 [1]. Due to the expanding population and rising risks of COPD risk factors such as tobacco use, dust, chemicals, burning biomass fuel, rising air pollution, etc., the burden of COPD is predicted to rise even more in the future [2].

Types of COPD

While there are other varieties of chronic obstructive pulmonary disease, emphysema and chronic bronchitis are the two most prevalent forms. COPD is usually caused by these two disorders. They often coexist and may differ in intensity between COPD patients. The lungs' tiny air sacs at the end of their airways rupture, which results in emphysema. Due to airway inflammation, chronic bronchitis causes a persistent cough that frequently produces phlegm [3]. Asthma is generally not a condition for COPD, but there are possibilities where one can have both asthma and COPD; this condition is known as asthma-COPD overlap syndrome (ACOS) [4].

Grades or stages of COPD

There are four stages of COPD. During the first stage, symptoms are modest and usually go unnoticed, with the exception of when someone is working out or exerting themselves. This stage's symptoms include a minor shortness of breath and a persistent dry cough. Stage two is the next step, and in this stage, the symptoms are worse than in stage one, including worsening shortness of breath, a persistent cough, and phlegm production. Phlegm's hue might alter during flare-ups. Symptoms in stage three are more severe than in stage two, especially in the mornings, and flare-ups occur more frequently. In some situations, people can report swollen ankles, feet, and legs. The last and final stage is stage four, in which the conditions are more exacerbated than in the other stages; difficulty in breathing, fast or irregular heartbeat, delirium, weight loss, pulmonary hypertension, etc. are possible [5].

Causes of COPD

There are several causes of COPD. The primary cause of or risk factor for COPD is long-term tobacco use. People who live in homes with poor ventilation and are exposed to the gases from burning fuel for cooking and heating usually develop COPD. COPD can also develop as a result of air pollution, occupational exposure to dust, smoke, or fumes, cigar smoking, secondhand smoke, pipe smoking, and other irritants. One of the main causes of COPD and other lung disorders is rising air pollution [6].

## Review

Methodology

We undertook a systematic search through PubMed and PubMed Central using keywords, such as "COPD", "metered-dose inhaler", "SMI", "DPI", "devices used", "nebulizers" and "ventilation gadgets," related to the topic (recent advances in the devices for the treatment of chronic obstructive pulmonary disease: a review). We additionally searched for key references from bibliographies of the relevant studies. 

**Figure 1:**
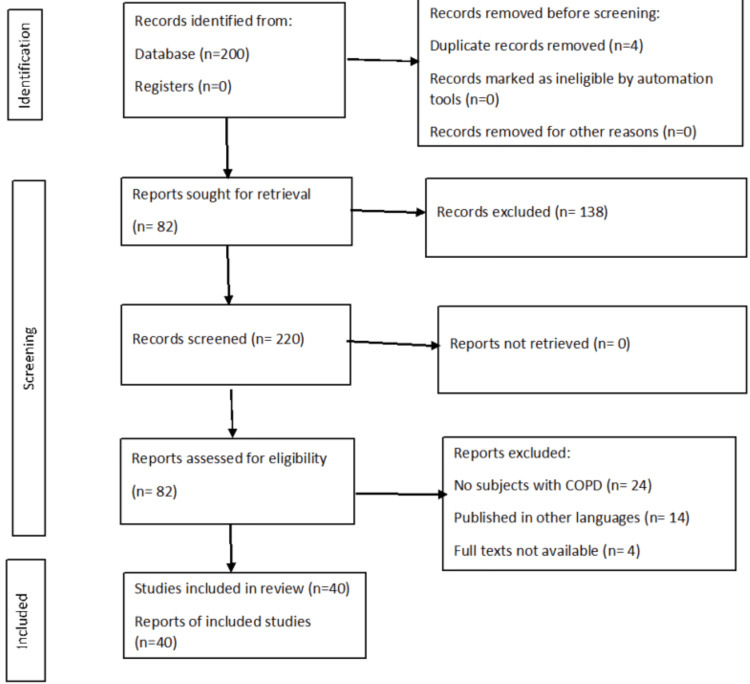
Prisma Chart The flowchart represents the methodology of the article on the recent advances in the devices for the treatment of chronic obstructive pulmonary disease

One reviewer independently monitored the retrieved studies against the inclusion criteria, in the beginning, based on the title and abstract and then on full texts. Another reviewer also reviewed approximately 20% of these studies to validate the inclusion of studies. Figure 1 shows the PRISMA flow chart.

COPD is a common respiratory disorder with significant morbidity and mortality. Despite its prevalence, it remains underdiagnosed most of the time and many patients do not receive adequate treatment for the disease until the symptoms of the disease advance [7].

Symptoms of COPD

Symptoms are generally considered the primary indications of a disease. So it is necessary to know the symptoms of COPD. Generally, individuals suffering from COPD may suffer from a cough that is dry or with phlegm, frequent respiratory infections, shortness of breath, or wheezing. They may experience fatigue or the inability to exercise. Also in some cases, they may experience chest pressure, loss of muscles, loss of weight, etc. To summarize, the symptoms include shortness of breath, wheezing, or a chronic cough [8].

Management of COPD

Management of a disease is very important for curing the disease. There are three types of management of COPD: lifestyle modification or lifestyle management of COPD, medical management of COPD, and device management of COPD. All these management types are explained below with a few examples in the following article.

Lifestyle modification in COPD patients

It is known that lifestyle modification is one of the most cost-effective strategies for self-management and secondary treatment of COPD. Hence, we should know the required lifestyle changes or modifications that are needed in COPD patients [9].

Quitting Smoking

Smoking is one of the most common causes of COPD or any other type of lung disease. It is the most effective strategy to reduce the progression or exacerbation of COPD. By quitting smoking, one can improve their lung capacity, oxygen intake, and breathing. Many programs are being organized by the government to stop smoking which can help individuals to quit smoking [10].

Staying Active

Exercise or in other words staying active is very effective as it strengthens muscles and improves endurance. It helps or trains the body to use oxygen more efficiently and reduces the shortness of breath during regular physical activities. Generally, gentle exercises are recommended, such as slow walking, gardening, etc., as they do not overexert the lungs [11]. Moreover, maintaining a healthy weight is also very effective in COPD. If an individual suffering from COPD is overweight, then weight loss can also help in reducing the symptoms of COPD because obesity can lead to breathing-related problems. A BMI of approximately 25 kg/m^2^ is considered beneficial for COPD patients. This can be achieved by staying active by doing small exercises like walking [12].

Getting Vaccinated

COPD is known to cause lung damage, so one can prevent further lung damage by avoiding infections like pneumonia and influenza by getting vaccinated for such infections as such conditions are the major cause of exacerbation, disease progression, and mortality in COPD. In this way, their lungs are protected from any kind of further damage that could be caused by such infections. So, vaccination is recommended for individuals suffering from COPD, in lifestyle modification [13].

Adapting the Work Environment

There are many individuals who are exposed to lung irritants at work. So, COPD patients who are frequently exposed to lung irritants, chemicals, fumes, etc. at work should consult with the supervisor regarding making changes to the work environment and should start wearing protective masks while working. One of the best approaches can be the removal of any kind of respiratory irritants and substituting the toxic agents with non-toxic agents. In the case where substitution is not possible, proper maintenance such as enclosing the industrial process and proper ventilation should be done [14].

Medical management of COPD

Exacerbation in COPD results in acute worsening of the respiratory tract, which needs to be managed properly. There are several pharmacological or medical therapies to treat COPD. The various classes of medication used for the treatment of COPD include long-acting beta 2-agonists (LABAs), long-acting muscarinic antagonists (LAMAs), and inhaled corticosteroids (ICS) [15].

Bronchodilators

Selective beta 2-agonists, anticholinergic antimuscarinic substances, and methylxanthines are all bronchodilators. Currently, they are the primary method of treating COPD. They often come as inhalers and ease the tension in the airway muscles. By using this technique, breathing rate is promoted, and coughing and breathlessness are alleviated. Patients with COPD are advised to take them. The two different types of bronchodilators are short-acting and long-acting. Albuterol, ipratropium, and levalbuterol are a few examples of short-acting bronchodilators. Long-acting bronchodilators include aclidinium, arformoterol, formoterol, indacaterol, tiotropium, salmeterol, and umeclidinium [16].

Antimuscarinic Drugs

They are used and administered similarly to bronchodilators. Muscarinic receptors M1 and M3 are primarily involved in this. Following the release of acetylcholine (ACh) by short postganglionic fibers, the M3 muscarinic receptors in airway smooth muscle cells are activated, leading to an increase in motility. Nicotinic and M1 muscarinic receptors are found in the respiratory tract's parasympathetic ganglia, and they are equally activated by vagal fibers. Stimulation of the M3 receptor leads to increased secretion from the bronchi. Cholinergic muscarinic receptors are the target of non-selective antimuscarinic bronchodilators. They have a major impact on airway blockage through submucosal gland cells' antagonistic M3 muscarinic receptors, which cause a decrease in basal and enhanced cholinergic parasympathetic activity, both of which reduce airway obstruction [17].

Theophylline

Theophylline, a methylxanthine, is one of the less expensive bronchodilators. Its bronchodilatory actions are brought about by both the competitive antagonism of adenosine receptors and the rather non-selective inhibition of cyclic nucleotide phosphodiesterases [18]. Typically, it is given intravenously, as sustained-release medication, or orally in typical ways. Hepatic metabolism mostly eliminates theophylline. Its elimination half-life usually ranges from eight to nine hours in adults and 3.5 hours in children. In cases of heart or liver failure, viral infection, old age, etc., its half-life is lengthened. Smokers, habitual drinkers, and other risk factors reduce the half-life. One of theophylline's disadvantages is its low therapeutic index. Anorexia, nausea, vomiting, insomnia, agitation, palpitations, and hypotension are theophylline's main adverse effects [19].

Antibiotics

Acute bronchitis, pneumonia, and other respiratory infections among others can exacerbate the symptoms of COPD. The main treatment for bouts of COPD worsening is antibiotics. It has been discovered that certain medicines, such as azithromycin, can stop COPD from getting worse. Antibiotics used as preventatives are useless in the winter [20].

Mucolytic Agents

Mucolytic agents like ambroxol, erdosteine, etc., compared to a placebo, cause a decrease in the incidence of exacerbations of chronic bronchitis [21].

Device therapy/management of COPD

COPD is a persistent respiratory condition portrayed via wind current impediment and trouble in relaxing. Its management frequently involves the utilization of various devices for further developing lung capability, reducing the side effects caused by medicine for COPD, and improving the patient’s general personal satisfaction. Some of these devices are described below:

Inhalers

Inhalers are an essential tool in COPD management, conveying medicines straightforwardly to the aviation routes. Some of the inhalers that are used are metered-portion inhalers (MDIs), dry powder inhalers (DPIs), and soft mist inhalers (SMIs). These gadgets have seen nonstop enhancements in drug conveyance proficiency, usability, and versatility. For example, few inhalers presently have hidden sensors that monitor usage and give inputs to patients and medical services suppliers [22].

Nebulizers

Nebulizers are devices that convert fluid medicine into a fine fog, which makes it simpler for the patients to breathe in the medication. Late advancements have focused on reducing treatment duration utilizing the nebulizer frameworks [23].

*Portable* *Oxygen* *Concentrators* (*POCs*)

They are more minimal and lightweight, hence permitting COPD patients to have more noteworthy versatility and autonomy. These devices give a ceaseless inventory of oxygen to people with extreme COPD and low blood oxygen levels [24].

*Mechanical* V*entilators*

Mechanical ventilators play a critical part in supporting breathing as well as keeping up satisfactory oxygen levels for serious COPD patients. Advancements in this area have led to more skilled and adaptable settings, decreasing the chance of confusion and improving tolerable outcomes [25].

*Noninvasive* V*entilation* (*NIV*) G*adgets*

NIV gadgets, for example, continuous positive airway pressure (CPAP) and bilevel positive airway pressure (BiPAP) machines, have become more agreeable and easy to use. They are in many cases used to treat intense intensifications of COPD and offer respiratory help without the requirement for obstructive intubation [26].

*Aviation* *Route* *Freedom* *Gadgets*

These gadgets assist with cleaning bodily fluid and flotsam and jetsam off of aviation routes, decreasing the gamble of diseases and further developing lung capability. Headway in this aspect has prompted more successful as well as patient-accommodating gadgets.

Recent advances in device therapy/management for COPD patients

**Figure 2:**
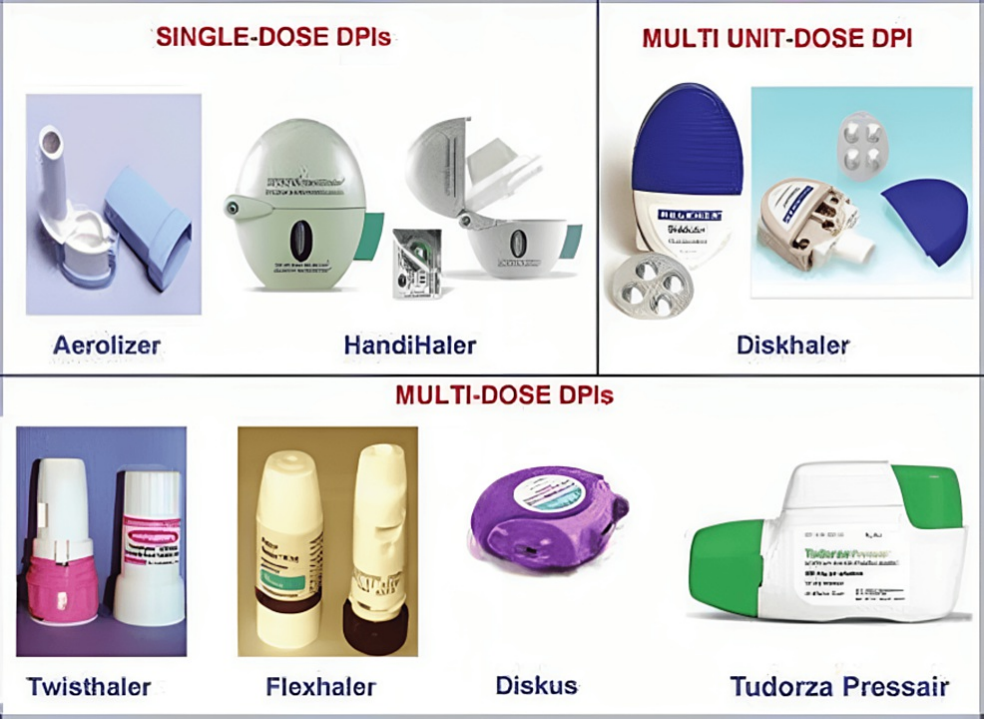
Dry powder inhalers The image is taken from an open-access website source [27]

Late advances in inward breathing treatments have zeroed in on upgrading drug conveyance, improving patient adherence, and creating novel details. These progressions include DPIs with further developed breath-incited components, empowering patients with compromised lung capability to actually get the endorsed portion. Figure 2 shows different types of DPIs which are used in COPD. Nifty inhalers outfitted with sensors and availability elements to follow medicine use remind patients to take their drugs properly on time and permit medical services suppliers to remotely screen adherence.

*Aspiratory* *Recovery* *Gadgets*

Late or current advances in this aspect have increased the availability of gadgets that support powerful aspiratory recovery, both in clinical settings and at home. A few critical headways include virtual reality (VR) and expanded reality (AR) frameworks that provide intelligence and interactions with objects, thus making aspiratory restoration more pleasant and stimulating for patients [28]. Wearable gadgets with attached coordinated sensors that screen respiratory boundaries as well as actual work during restoration, thus giving important information to patients and medical care suppliers, are another advancement in the devices for COPD patients [29].

*Remote* *Observing* *Frameworks*

Advancements in this area have been found as a unique advantage in the administration of persistent illnesses like COPD. Ongoing advances in remote checking for COPD include remote painless gadgets that monitor fundamental signs, oxygen immersion levels, and respiratory boundaries, communicating continuous information to medical services suppliers. Integration of computerized reasoning and AI calculations into remote observing frameworks empowers early discovery of COPD intensifications because of minor modifications in information frameworks [30].

Telemedicine

Telemedicine refers to the use of information and communications technology to deliver health services, particularly in situations when access to care is hampered by distance. The use of telemedicine is favored by a number of significant features, including real-time audio and video communication that links doctors and patients at the same time, information exchange that does not require both individuals to be present at the same time, and the use of a variety of wearable devices, accessories, sensors, etc. These characteristics guarantee that patients' sense of security, health awareness, and medication adherence are all positively impacted in a way that saves both time and money [31].

Single Inhaler Triple-Drug Therapy

**Figure 3:**
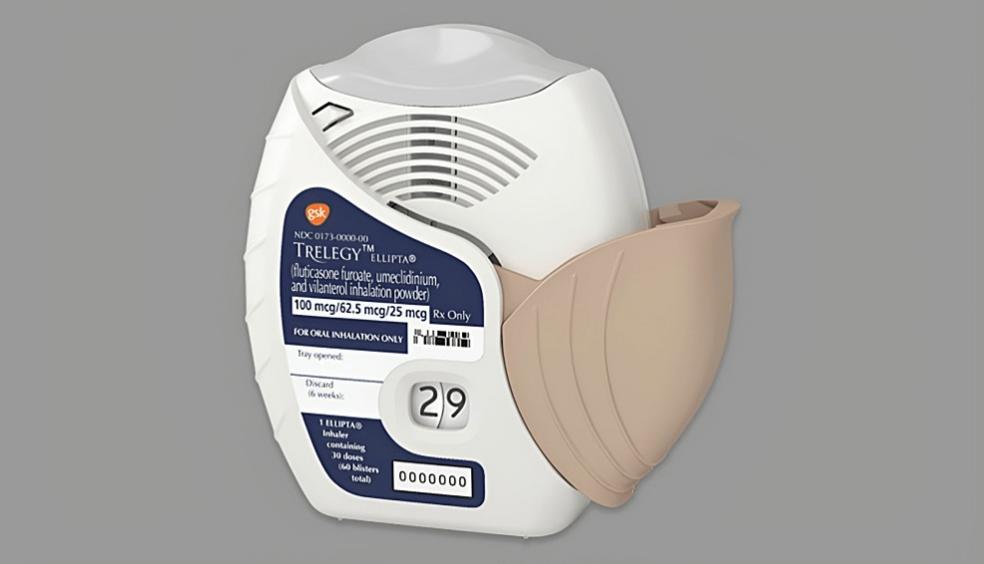
New inhaler that combines three drugs together The image is taken from an open-access website source [33]

A novel advancement has been discovered recently: the ability to combine three medications into one inhaler. It involves the combination of fixed ICS/LABA with LAMA or vice versa. This method prevented the overuse of medications and exacerbation risk while maintaining a high level of safety and health. It also demonstrated improved lung function when compared to ICS/LABA or independent LAMA treatment [32]. Figure 3 shows the newly developed inhaler that combines three medicines, becoming a triple-drug inhaler.

Advantages

The ongoing advances in devices used for the therapy of COPD bring various benefits and advantages that preferentially work on the administration as well as on the personal satisfaction of patients. Some of the benefits of these advances in devices are mentioned in the following paragraph.

Current inward breath gadgets, like excellent inhalers and further developed DPIs, guarantee more exact and viable medication conveyance to the lung [22]. This designated conveyance upgrades the helpful viability of medicines, promoting better side effects control and decreased aftereffects. Smart inhalers and remote observing frameworks enhance drug adherence by reminding patients to take their assigned portions on time and follow their medicine use. The ability to provide mixed treatments in a solitary inhaler can enable treatment regimens customized for individual COPD patients. This approach ensures that the patient gets the right blend of prescriptions, enhancing their treatment and possibly lessening the number of endorsed inhalers they need to utilize.

Remote observing frameworks which are outfitted with sensors and AI calculations permit nonstop observance of the patients' wellbeing status. These frameworks can recognize mild changes in respiratory boundaries, taking into account early detection of intensifications or worsening side effects [34]. The utilization of wearable gadgets and versatile applications engages COPD patients to take part in their treatment effectively. Patients can conveniently follow their side effects, screen imperative signs, and access medical information, which encourages self-administration and provides a superior comprehension of their condition [35]. These advances can also possibly decrease the financial weight of COPD patients on medical services frameworks as fewer hospitalization and trauma center visits mean lower medical care costs and better-developed asset components. Overall, these advancements give patients a better command of their illness and engage them to effectively participate in their treatment. Through superior medication conveyance, adherence, and early identification, these devices can essentially work on the personal satisfaction of COPD patients and diminish the weight of the infection on the medical care framework [36].

Challenges faced in device therapy

While late advances in device therapy for COPD provide various benefits, there are additionally some possible burdens and difficulties associated with their utilization. These challenges should be considered for an extensive comprehension of the effect of these developments. Various challenges faced have been mentioned in the following paragraph.

Many high-level COPD treatment devices, like smart inhalers and remote checking frameworks, may accompany greater expenses in contrast to customary inhalers or non-associated devices. These costs could be a hindrance to access for certain patients, especially those without satisfactory protection inclusion or restricted monetary assets. Another challenge faced is that of technological barriers. COPD fundamentally influences more seasoned grown-ups, and some patients may not be familiar with or happy with utilizing complex innovations. This can prevent the successful utilization of smart inhalers, portable applications, or wearable gadgets [37].

Remote checking frameworks and smart devices gather and share well-being information, so there could be worries about the security and protection of this data. Guaranteeing information protection and consistency with security guidelines are fundamental. Dependence on technology is another challenge faced. While cutting-edge gadgets provide various advantages, many patients are growing dependent on technology or innovation to treat their illnesses. Over-reliance on innovation may lead to ignorance of other vital parts of COPD care, for example, way of life changes and adherence to medicine. Also, maintenance of these devices is required. Assuming that these devices experience glitches, programming issues, or availability issues, it might lead to patient dissatisfaction and affect the effectiveness of therapy [38]. Increased dependence on remote checking and virtual communications might cause decreased personal connections among patients and medical care suppliers. Special interactions and sympathetic correspondence are fundamental in medical services, and the lack of in-person discussions could affect the nature of the care given.

Despite these challenges, the continuous refinement of cutting-edge COPD devices, tending to expected difficulties, and developing greater transparency could further enhance their advantages and contribute to better COPD management and patient results. It is important or necessary for medical care suppliers, producers, etc. to defeat these challenges or drawbacks of devices and ensure that these advancements are open, powerful, and easy to use for all COPD patients [39].

## Conclusions

COPD is a pervasive and incapacitating respiratory condition that influences a large number of individuals around the world. Ongoing advances in the devices for the treatment or therapy of COPD are a huge step in the right direction in the management of this persistent respiratory condition. These advancements provide many advantages that can possibly improve health outcomes, improve illness, and lessen the burden of COPD on people and medical care frameworks. These devices can reduce the side effects and aftereffects caused by medicines. However, there are some challenges faced in the device therapy of COPD, such as information security concerns, dependence on innovation, etc. Resolving these issues is essential to guaranteeing full access to these advancements and maximizing their impact on the disease. Various new advances are ongoing to eradicate these challenges. All things considered, the new advances in devices for COPD treatment have a significant impact on how this illness is treated.

## References

[1] Vogelmeier CF, Román-Rodríguez M, Singh D, Han MK, Rodríguez-Roisin R, Ferguson GT (2020). Goals of COPD treatment: focus on symptoms and exacerbations. Respir Med.

[2] Halpin DM, Criner GJ, Papi A (2021). Global initiative for the diagnosis, management, and prevention of chronic obstructive lung disease. The 2020 GOLD science committee report on COVID-19 and chronic obstructive pulmonary disease. Am J Respir Crit Care Med.

[3] (2023). Types of COPD | ATrain Education. https://www.atrainceu.com/content/1-types-copd.

[4] (2023). Types of COPD: symptoms, treatments, and more. https://www.healthline.com/health/copd/types-of-copd.

[5] (2023). Recognizing the 4 stages of COPD.. https://www.bayfronthealth.com/content-hub/recognizing-the-4-stages-of-copd.

[6] López-Campos JL, Tan W, Soriano JB (2016). Global burden of COPD. Respirology.

[7] Ferrera MC, Labaki WW, Han MK (2021). Advances in chronic obstructive pulmonary disease. Annu Rev Med.

[8] Labaki WW, Rosenberg SR (2020). Chronic obstructive pulmonary disease. Ann Intern Med.

[9] Yan R, Wang Y, Bo J, Li W (2017). Healthy lifestyle behaviors among individuals with chronic obstructive pulmonary disease in urban and rural communities in China: a large community-based epidemiological study. Int J Chron Obstruct Pulmon Dis.

[10] Tashkin DP (2015). Smoking cessation in chronic obstructive pulmonary disease. Semin Respir Crit Care Med.

[11] Woo J (2023). Relationships among diet, physical activity and other lifestyle factors and debilitating diseases in the elderly. Eur J Clin Nutr.

[12] Gologanu D, Ionita D, Gartonea T, Stanescu C, Bogdan MA (2014). Body composition in patients with chronic obstructive pulmonary disease. Maedica (Bucur).

[13] Froes F, Roche N, Blasi F (2023). Pneumococcal vaccination and chronic respiratory diseases. Int J Chron Obstruct Pulmon Dis.

[14] Bourbeau J, Nault D, Dang-Tan T (2004). Self-management and behaviour modification in COPD. Patient Educ Couns.

[15] Celli BR, Wedzicha JA (2019). Update on clinical aspects of chronic obstructive pulmonary disease. N Engl J Med.

[16] Montuschi P (2006). Pharmacological treatment of chronic obstructive pulmonary disease. Int J Chron Obstruct Pulmon Dis.

[17] Wolstenholme RJ (2014). Replacing β agonists with antimuscarinic drugs in patients with COPD. BMJ.

[18] Barnes PJ (2003). Theophylline: new perspectives for an old drug. Am J Respir Crit Care Med.

[19] Barnes PJ, Pauwels RA (1994). Theophylline in the management of asthma: time for reappraisal?. Eur Respir J.

[20] MacNee W, Calverley PM (2003). Chronic obstructive pulmonary disease • 7: management of COPD. Thorax.

[21] Poole PJ, Black PN (2003). Mucolytic agents for chronic bronchitis or chronic obstructive pulmonary disease. Cochrane Database Syst Rev.

[22] Bourbeau J, Bafadhel M, Barnes NC (2021). Benefit/risk profile of single-inhaler triple therapy in COPD. Int J Chron Obstruct Pulmon Dis.

[23] Barjaktarevic IZ, Milstone AP (2020). Nebulized therapies in COPD: past, present, and the future. Int J Chron Obstruct Pulmon Dis.

[24] (2023). COPD devices: nebulizers and Inhalers (MDI and DPI). https://www.webmd.com/lung/copd/how-copd-devices-work.

[25] Corrado A, Ginanni R, Villella G (2004). Iron lung versus conventional mechanical ventilation in acute exacerbation of COPD. Eur Respir J.

[26] Majorski DS, Magnet FS, Thilemann S, Schmoor C, Windisch W, Schwarz SB (2021). Portable NIV for patients with moderate to severe COPD: two randomized crossover trials. Respir Res.

[27] (2023). Dry powder inhalers (DPIs) currently available in the United States. https://www.researchgate.net/figure/Dry-powder-inhalers-DPIs-currently-available-in-the-United-States-categorized-by_fig3_258041169.

[28] Reiners D, Davahli MR, Karwowski W, Cruz-Neira C (2021). The combination of artificial intelligence and extended reality: a systematic review. Frontiers in Virtual Reality.

[29] Celli BR, Singh D, Vogelmeier C, Agusti A (2022). New perspectives on chronic obstructive pulmonary disease. Int J Chron Obstruct Pulmon Dis.

[30] Taylor A, Lowe DJ, McDowell G, Lua S, Burns S, McGinness P, Carlin CM (2021). Remote-management of COPD: evaluating implementation of digital innovation to enable routine care (RECEIVER): protocol for a feasibility and service adoption observational cohort study. BMJ Open Respir Res.

[31] Fekete M, Fazekas-Pongor V, Balazs P, Tarantini S, Nemeth AN, Varga JT (2021). Role of new digital technologies and telemedicine in pulmonary rehabilitation: smart devices in the treatment of chronic respiratory diseases. Wien Klin Wochenschr.

[32] Vanfleteren L, Fabbri LM, Papi A, Petruzzelli S, Celli B (2018). Triple therapy (ICS/LABA/LAMA) in COPD: time for a reappraisal. Int J Chron Obstruct Pulmon Dis.

[33] (2023). Trelegy Ellipta Inhaler. https://cdn.aarp.net/content/dam/aarp/health/drugs_supplements/2017/09/1140-trelegy-ellipta-inhaler.jpg.

[34] Morimoto Y, Takahashi T, Sawa R (2022). Web portals for patients with chronic diseases: scoping review of the functional features and theoretical frameworks of telerehabilitation platforms. J Med Internet Res.

[35] Buekers J, Arbillaga-Etxarri A, Gimeno-Santos E, Donaire-Gonzalez D, Chevance G, Aerts JM, Garcia-Aymerich J (2023). Heart rate and oxygen uptake kinetics obtained from continuous measurements with wearable devices during outdoor walks of patients with COPD. Digit Health.

[36] Quach S, Benoit A, Oliveira A, Packham TL, Goldstein R, Brooks D (2023). Features and characteristics of publicly available mHealth apps for self-management in chronic obstructive pulmonary disease. Digit Health.

[37] Meghji J, Mortimer K, Agusti A (2021). Improving lung health in low-income and middle-income countries: from challenges to solutions. Lancet.

[38] Janjua S, Carter D, Threapleton CJ, Prigmore S, Disler RT (2021). Telehealth interventions: remote monitoring and consultations for people with chronic obstructive pulmonary disease (COPD). Cochrane Database Syst Rev.

[39] Jenkins C (2016). Successes and challenges of COPD management in Australia: reflections on the past and future. Lancet Respir Med.

